# Detection of time of birth and cord clamping using thermal video in the delivery room

**DOI:** 10.3389/fped.2024.1342415

**Published:** 2024-08-14

**Authors:** Vilde Kolstad, Jorge García-Torres, Sara Brunner, Anders Johannessen, Elizabeth Foglia, Hege Ersdal, Øyvind Meinich-Bache, Siren Rettedal

**Affiliations:** ^1^ Department of Simulation-Based Learning, Stavanger University Hospital, Stavanger, Norway; ^2^ Department of Electrical Engineering and Computer Science, University of Stavanger, Stavanger, Norway; ^3^ Laerdal Medical AS, Stavanger, Norway; ^4^ Division of Neonatology, Perelman School of Medicine, University of Pennsylvania, Philadelphia, PA, United States; ^5^ Faculty of Health Sciences, University of Stavanger, Stavanger, Norway

**Keywords:** birth detection, time of birth, newborn resuscitation timelines, resuscitation guidelines, cord clamping, thermal video, delivery room

## Abstract

**Introduction:**

Newborn resuscitation algorithms emphasize that resuscitation is time-critical, and all algorithm steps are related to the time of birth. Infrared thermal video has the potential to capture events in the delivery room, such as birth, cord clamping, and resuscitative interventions, while upholding the privacy of patients and healthcare providers.

**Objectives:**

The objectives of this concept study were to (i) investigate the technical feasibility of using thermal video in the delivery room to detect birth and cord clamping, and (ii) evaluate the accuracy of manual real-time registrations of the time of birth and cord clamping by comparing it with the accuracy of registrations abstracted from thermal videos.

**Methods:**

An observational study with data collected at Stavanger University Hospital, Norway, from September 2022 to August 2023. The time of birth and cord clamping were manually registered on a portable tablet by healthcare providers. Thermal cameras were placed in the delivery rooms and operating theatre to capture births. Videos were retrospectively reviewed to determine the time of birth and cord clamping.

**Results:**

Participation consent was obtained from 306 mothers, of which 195 births occurred in delivery rooms or an operating theatre with a thermal camera installed. We excluded 12 videos in which no births occurred. Births were detectable in all 183 (100%) thermal videos evaluated. There was a median (quartiles) of 1.8 (0.7, 5.4) s deviation in the manual registrations of the times of births relative to those abstracted from thermal videos. Cord clamping was detectable in 173 of the 183 (95%) thermal videos, with a median of 18.3 (3.3, 108) s deviation in the manual registrations of the times of cord clampings relative to those abstracted from thermal videos.

**Conclusion:**

Recognizing the time of birth and cord clamping from thermal videos is technically feasible and provides a method for determining when resuscitative events occur.

## Introduction

Newborn resuscitation is a time-critical and frequently occurring event. Between 3% and 10% of newborns do not breathe spontaneously and need resuscitation with positive pressure ventilation (PPV) at birth (1–4). Newborn resuscitation algorithms are time-dependent, and all steps in the algorithms are relative to the time of birth (TOB). Newborns not breathing effectively after drying and tactile stimulation, should have their heart rate (HR) assessed and PPV initiated within 60 s after birth (3, 4). Although delayed cord clamping (CC) for at least 30–60 s is recommended as standard care (3, 4), most newborns thought to need resuscitation have the cord clamped and cut early for transfer to the resuscitation table.

Documentation of newborn resuscitations is often inaccurate or incomplete, in most cases performed retrospectively, and based on recall (5–7). Technological advances with automated capture and synchronization of data from newborn resuscitations have recently enabled the evaluation of the sequences, timing, and duration of resuscitative interventions, as well as HR responses to treatment (8–12). All interventions are contextualized relative to the TOB, but an automated and accurate method for registering the TOB for newborns is not available.

Infrared thermal video is a non-invasive technology that can capture and generate images using the thermal radiation emitted and reflected by objects. The higher the temperature, the more radiation is emitted. It is increasingly used in healthcare, for example, for detecting temperature anomalies during disease outbreaks (13, 14). For newborns, thermal imaging has been explored in the evaluation of clinical conditions such as thermoregulation and respiratory rate monitoring (15–18). In addition, thermal imaging has been used for the detection of pathological conditions that include inflammatory responses such as necrotizing enterocolitis (19). An ongoing research project, NewbornTime, aims to explore how artificial intelligence (AI) and thermal video can be leveraged for the automated detection of birth and delivery room resuscitative interventions (20, 21). Given that the skin temperature of the newborn immediately after birth is higher than the skin temperature of the mother, AI-based algorithms can use thermal video to automatically detect the TOB while upholding the privacy of all persons involved.

The objectives of this concept study were to (i) investigate the technical feasibility of using thermal cameras in the delivery rooms to detect the TOB and time of CC, and (ii) evaluate the accuracy of real-time manual registrations of the TOB and time of CC by comparing it with the accuracy of registrations abstracted from thermal videos.

## Methods

### Study design and setting

This study is a prospective observational study with data collected at Stavanger University Hospital, Norway, from 7 September 2022 to 14 August 2023. This hospital is a tertiary level hospital with 4,000 births annually. Vaginal births take place in the delivery unit (84%) and caesarean sections in the operating theatre (16%). Overall, 3.6% of newborns receive resuscitation with PPV at birth (1).

### Data collection and equipment

**Figure 1:**
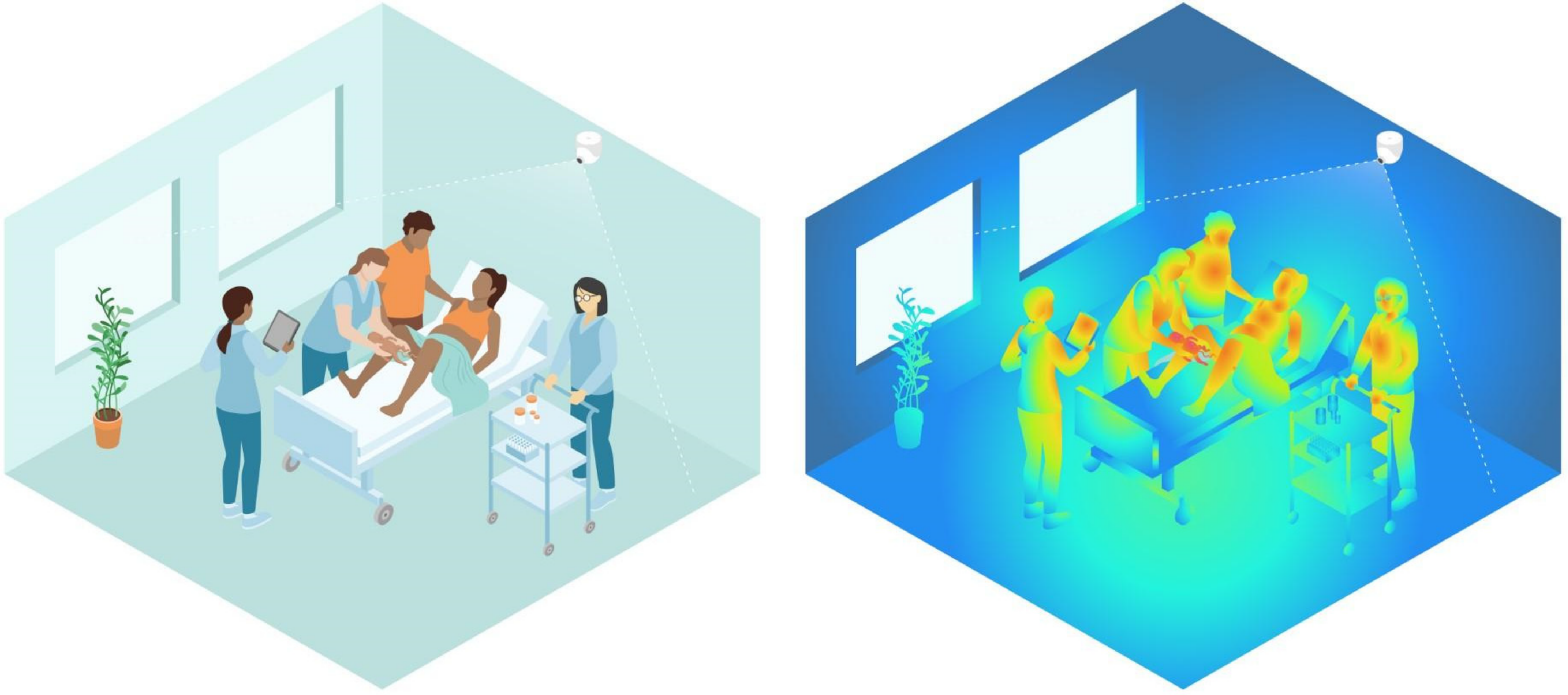
Illustration of a thermal camera placed above the head of the bed, capturing temperature differences in the delivery room. The application of thermal video plays a crucial role in protecting the privacy of mothers and healthcare providers. Illustrations made with Adobe Illustrator; used with permission from Laerdal Medical AS, Fredrik Kleppe.

Thermal videos from birth were captured using passive thermal cameras (an Mx-O-SMA-TPR079 thermal sensor connected to an Mx-S16B camera, Mobotix), placed on the ceiling behind the hospital bed in eight of 14 delivery rooms and one operating theatre, as illustrated in Figure 1. Recordings were started when any pixel in the video frame was above 30°C.

**Figure 2:**
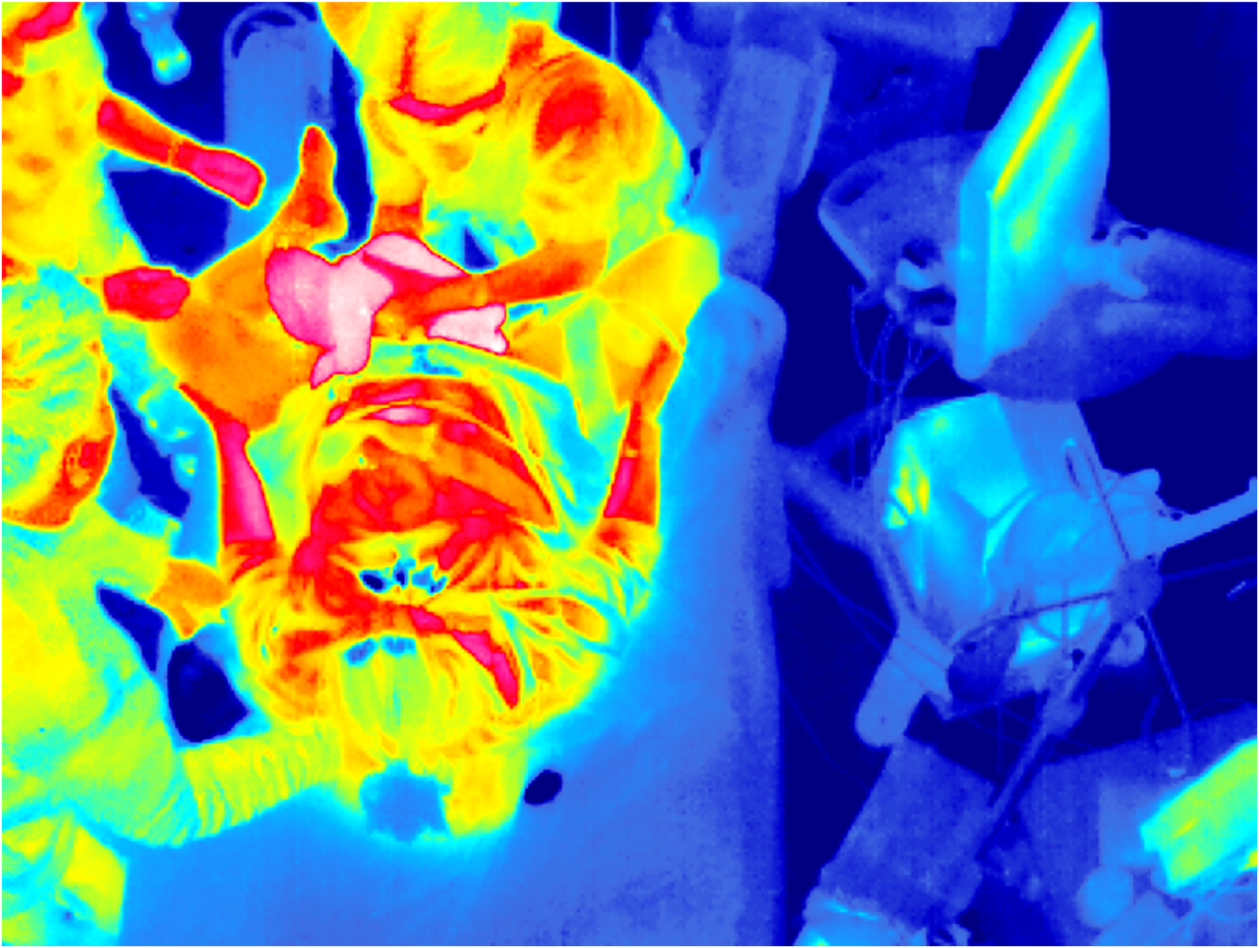
The time of birth recorded by a thermal camera in the delivery room. The contour of the newborn is clearly visible in “pink” due to a higher skin temperature than other humans and objects.

**Figure 3:**
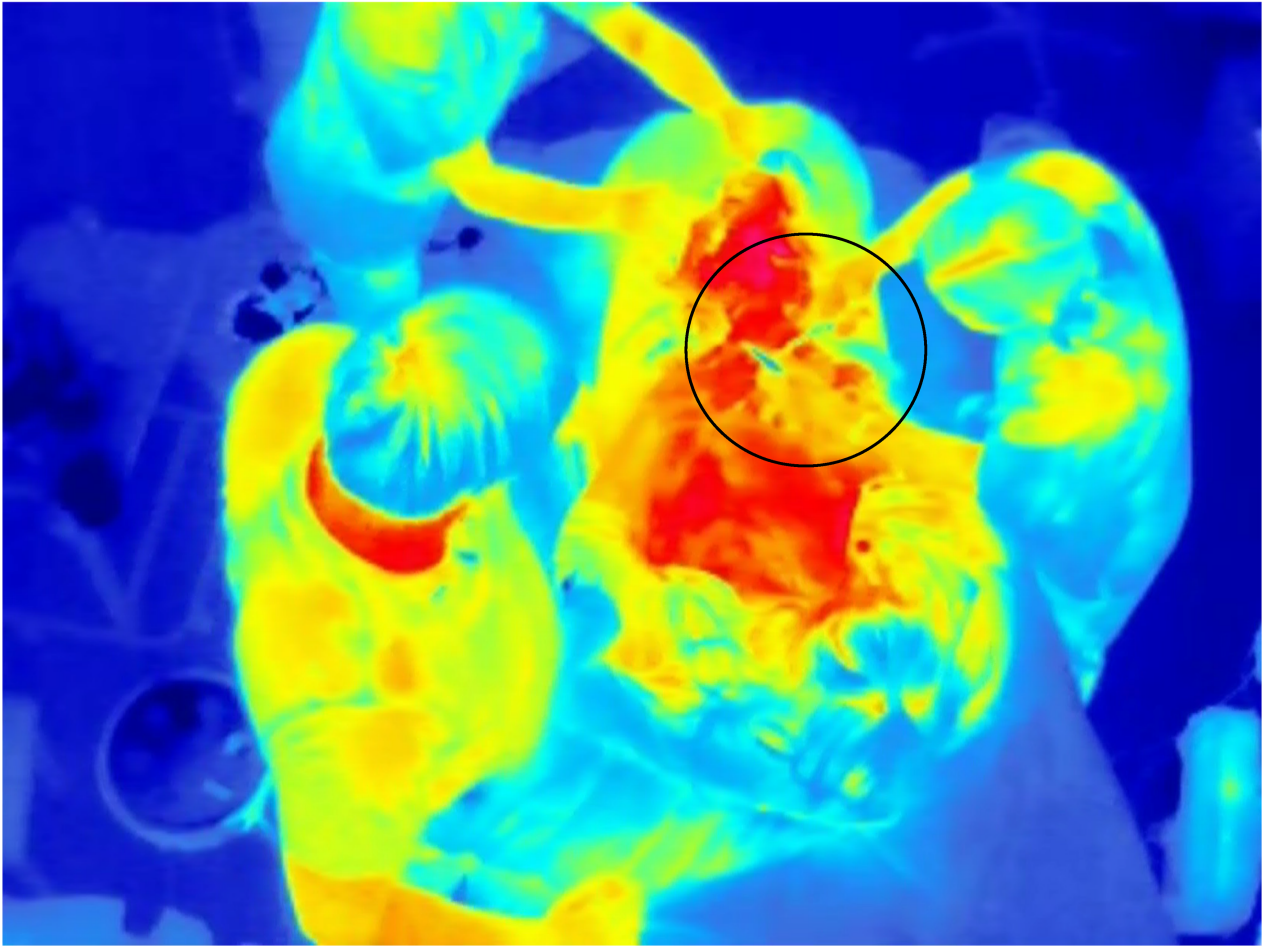
Time of cord clamping recorded by a thermal camera in the delivery room; the region of interest is indicated by a circle. Sequential video frames make it easier to recognize the movements of healthcare providers clamping and cutting the umbilical cord, compared with a single thermal image as illustrated here.

Thermal radiation captured by the sensor is presented as intensity values (grey scale). To improve visualization, a colour map can be applied to better represent and understand the thermal data, as shown in Figures 2, 3.

Manual registration of the TOB and time of CC was collected by the nurse assistants who pressed “baby born” and “cord clamped” in the Liveborn application designed for registrations of events after birth (Laerdal Medical AS, Stavanger, Norway). Following the activation of birth in the Liveborn application, a thermal video without audio was automatically produced by recording and storing a period of ±15 min centred around the manually registered TOB. Patient characteristics were extracted from the electronic medical records.

### Data annotation and definitions

Thermal videos were reviewed and the TOB and time of CC were annotated by independent researchers (VK and JG-T) using the ELAN 5.8 tool (The Language Archive, Nijmegen, The Netherlands). The TOB was defined as the time when we observed that the newborn was distinctly clear of the mother’s perineum or by identifying movements of the healthcare providers clearly indicating the birth of the newborn. The time of CC was defined as when we observed the clamp being placed on the umbilical cord. After the annotations of thermal videos had been completed, a comparison of absolute differences from the manual registrations was performed.

### Inclusion and exclusion criteria

Mothers were approached when research assistants were available to obtain consent at routine ultrasound screening during pregnancy, or at admission for labour. We included all newborns for whom the TOB was manually registered in the Liveborn application and the birth occurred in a delivery room with a thermal camera installed. The birth was considered not detectable if an exact TOB was not visible in the video due to obstruction by people or objects and the movements of healthcare providers did not clearly indicate that the baby was born. Videos were excluded if no mother gave birth in the video.

### Statistical analysis

Data analysis was conducted using Python 3. Absolute differences in the TOB and time of CC were estimated by subtracting the manual registrations from thermal annotations and obtaining the absolute value. Further assessment of the median and quartiles and evaluation of the distribution of data were then performed.

### Ethical considerations

This study is part of the NewbornTime research project, with ethical approval from the Norwegian Regional Ethical committee, region west, Norway (222455), and the Norwegian Agency for Shared Services in Education and Research (identifier 816989). Informed consent was obtained from all mothers included in the study. Healthcare providers could refrain from participation, in which case, the video was deleted.

## Results

**Figure 4:**
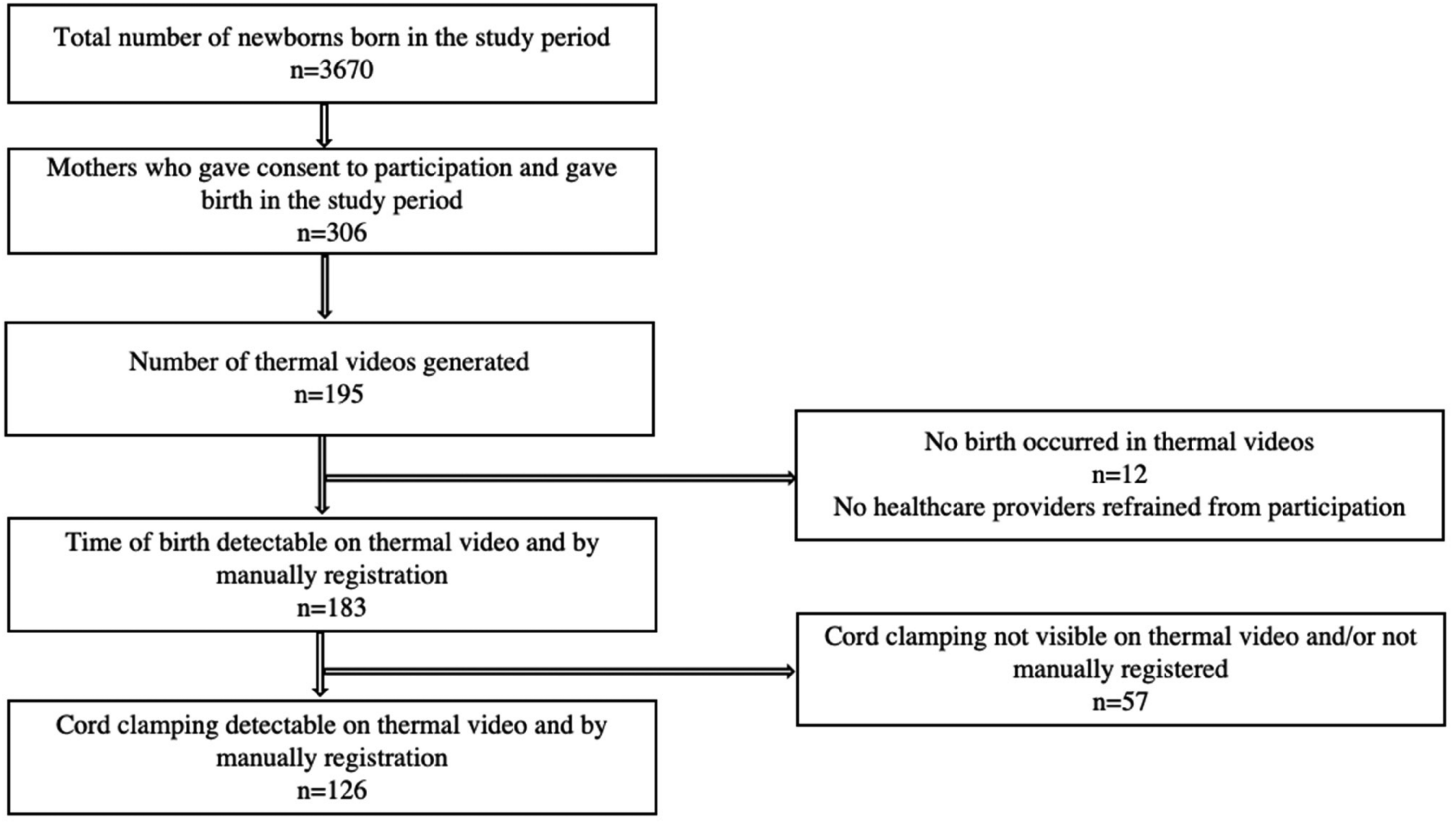
Inclusion of study patients.

During the study period, 3,670 newborns were born at the hospital. There were 306 births registered with consent to participate, of which 195 births occurred in a delivery room or in the operating theatre with a thermal camera installed. We excluded 12 videos in which no mother gave birth. Among 183 videos including a birth, 144 (79%) newborns were delivered vaginally and 39 (21%) by caesarean section. Among the vaginal births, 5.5% of the mothers gave birth in a hands and knees position, 6.1% in a side-lying position, and the rest in the supine position. PPV was provided to 7 (3.8%) of the newborns after birth. The median (quartiles) gestational age was 40 (39, 40) weeks and the median (quartiles) birthweight was 3,565 (3,210, 3,910) g. The enrolment of patients is illustrated in Figure 4.

Birth was detectable in all 183 (100%) thermal videos. In 177 (97%) cases, the TOB was detected by direct identification of the newborn based on infrared radiation. In the remaining 6 (3%) videos, the TOB was identified by recognizable movements of healthcare providers or mothers indicating the newborn had been born.

Time of CC was detectable by thermal video in 173 of 183 (97%) newborns. CC was detectable in 98% (141/144) of vaginal births and 82% (32/39) of caesarean sections. Delayed CC after 60 s occurred in 76% (132/173) of births; of these, 95% (125/132) occurred in the delivery room and 5% (7/132) in the operating theatre. Early CC before 60 s occurred in six of seven newborns receiving PPV, and of these, three were caesarean sections. In the 10 thermal videos in which the time of CC could not be detected, three newborns were born vaginally and CC occurred beyond the duration of the video recording. The remaining seven cases were caesarean sections, in which humans or objects blocked the field of vision.

The absolute difference in time between manual registration in real-time and registrations from thermal videos is presented in Table 1. The absolute difference in registration of the TOB was 1.8 (0.7, 5.4) s. In 74% of births, the absolute difference in the TOB was <5 s, and in 89% of births the absolute difference in the TOB was <10 s.

**Table 1:** The absolute difference in seconds for the TOB and time of CC between thermal video annotation and manual registration in the labour room, operating theatre, and overall.

	Labour room TOB (*n* = 144) CC (*n* = 105)			Operating theatre TOB (*n* = 39) CC (*n* = 21)			Total TOB (*n* = 183) CC (*n* = 126)		
	P25	P50	P75	P25	P50	P75	P25	P50	P75
TOB	0.7	1.8	4.9	0.7	1.8	6.1	0.7	1.8	5.4
CC	3.3	17.8	88.6	4.1	27.2	137	3.3	18.3	108

P is the percentile: P50 corresponds to the median, and the interval (P25, P75) corresponds to quartiles.

**Figure 5:**
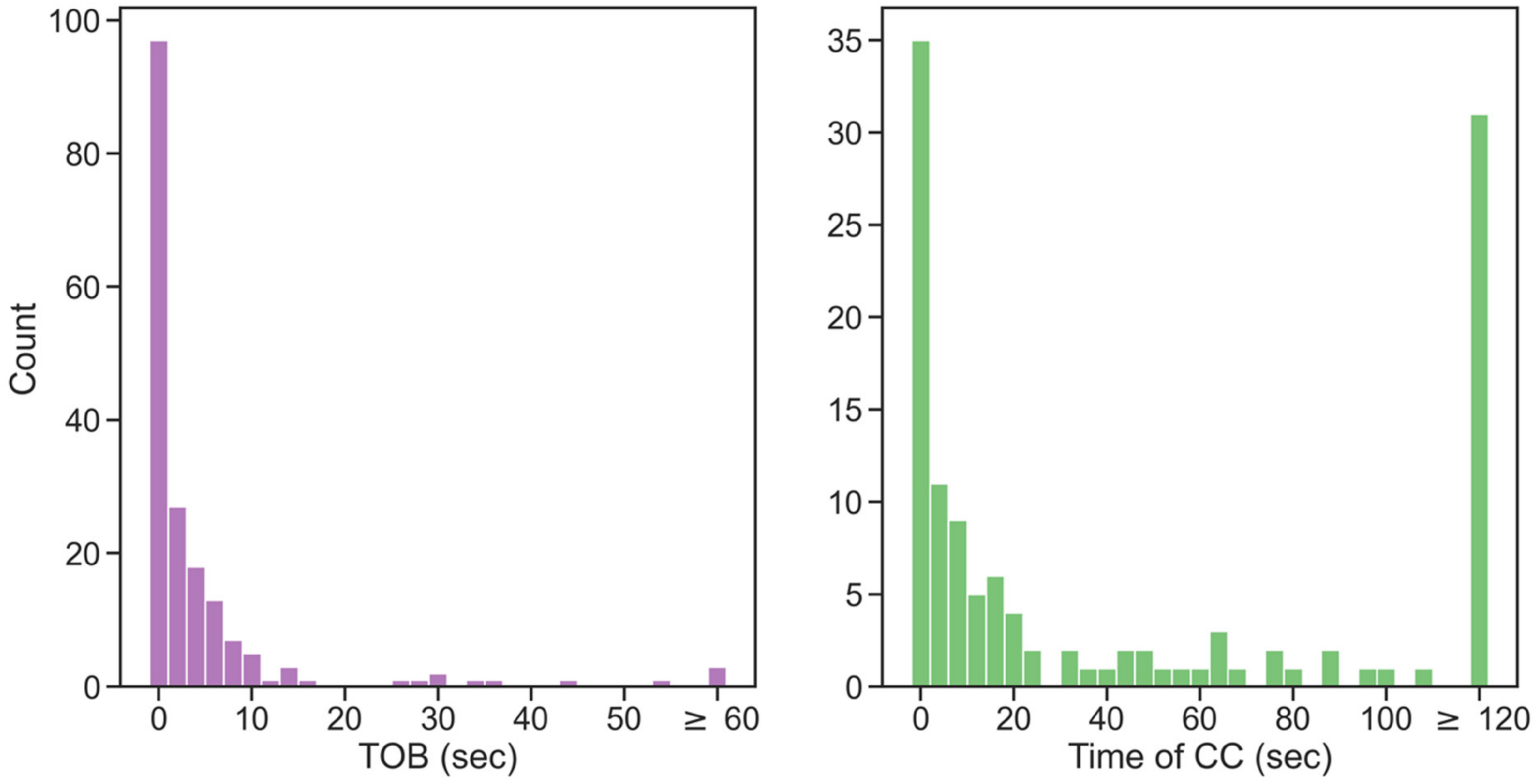
Histograms showing the absolute difference between thermal video annotation and the manual registration of the TOB and time of CC.

Manual registration of CC was missing in 52 cases. Five videos had a missing time of CC in both the thermal video and manual registration, leaving a total of 126 cases available for comparison. The absolute difference in the registration time of CC was 18.3 (3.3, 108) s. Histograms of the absolute difference are shown in Figure 5.

## Discussion

In this concept study, we demonstrated the feasibility of using thermal videos to identify key delivery room events and interventions. Even with a single thermal camera installed in the delivery room and different birthing positions, identifying the TOB and CC was technically feasible. The TOB and CC were visible in 100% and 97% of cases, respectively.

Accurate TOB registration is crucial as newborn resuscitative interventions are related to birth and actions are considered time-critical. In a study in a low-resource setting, the risk of newborn death or prolonged admission increased by 16% for every 30 s delay in the time to start ventilation after birth (22). Although the same correlation has been difficult to demonstrate in high-resource settings, resuscitation algorithms all start at the time when the newborns are born.

The precision of manual TOB registrations documented by nurse assistants was high in our setting, with a median of 1.8 s absolute difference between manual and video-based registrations. However, in 11% of births, there was more than a 10 s difference between manually registered TOBs and registrations from thermal videos, which suggests that alternate methods for detecting the TOB may improve the accuracy of documentation. It is possible that this technology could be further improved by employing AI to automatically detect the TOB on thermal videos; future studies will address this topic.

There was a larger variance in the precision of manual registrations for the time of CC. We speculate that this was caused by healthcare providers considering it less critical to register CC accurately, or that patient care required their attention. It was more difficult to detect the accurate time of CC in thermal videos from the operating theatre due to healthcare providers and objects blocking the view. In future studies, cameras should be placed more optimally in the ceiling above the operating field.

The use of thermal videos in the delivery room opens up the possibility of objectively evaluating the incidence, sequences, timing, and duration of obstetrical and newborn management. Thermal videos can be exploited to evaluate resuscitative interventions such as tactile stimulation of the newborn, palpation or auscultation of the newborn HR, suction of the airways, and maternal-newborn skin-to-skin time. Although optic video is increasingly used for research and quality improvement initiatives, thermal video has the vast advantage of protecting the privacy of patients and healthcare providers. Furthermore, using thermal videos to automatize data capture may allow healthcare providers to reallocate their attention from documenting resuscitation to supporting patient care.

Our research group is currently performing a study analysing heart rate responses to tactile stimulation immediately after birth using thermal videos and dry-electrode ECG technology. In future studies, the benefits of combining thermal videos and AI will be explored to develop a video-based system to automatically register timelines of resuscitation events from birth.

A limitation of the current study is the low inclusion rate among newborns born at the hospital, as recruitment was only performed when research assistants were available to take consent. Therefore, this study only reports on the technical feasibility of detecting the TOB and time of CC. Finally, caesarean births were somewhat overrepresented in our cohort.

## Conclusion

Recognizing the TOB and CC from thermal videos is technically feasible and can accurately measure when critical delivery room events occur. By comparing the manual registration of the TOB and CC with thermal videos, we found a median absolute difference of 2 and 18 s, respectively. The use of thermal birth videos may facilitate more precise timelines of delivery room interventions during and after birth while upholding the privacy of patients and healthcare providers.

## Data Availability

The original contributions presented in the study are included in the article/Supplementary Material, further inquiries can be directed to the corresponding author.

## References

[1] Bjorland PA Oymar K Ersdal HL Rettedal SI. Incidence of newborn resuscitative interventions at birth and short-term outcomes: a regional population-based study. BMJ Paediatr Open. (2019) 3(1):e000592. 10.1136/bmjpo-2019-000592 31909225 PMC6936999

[2] Niles DE Cines C Insley E Foglia EE Elci OU Skare C Incidence and characteristics of positive pressure ventilation delivered to newborns in a US tertiary academic hospital. Resuscitation. (2017) 115:102–9. 10.1016/j.resuscitation.2017.03.035 28411062

[3] Madar J Roehr CC Ainsworth S Ersdal H Morley C Rudiger M European resuscitation council guidelines 2021: newborn resuscitation and support of transition of infants at birth. Resuscitation. (2021) 161:291–326. 10.1016/j.resuscitation.2021.02.014 33773829

[4] Wyckoff MH Greif R Morley PT Ng KC Olasveengen TM Singletary EM International consensus on cardiopulmonary resuscitation and emergency cardiovascular care science with treatment recommendations: summary from the basic life support; advanced life support; pediatric life support; neonatal life support; education, implementation, and teams; and first aid task forces. Resuscitation. (2022) 2022(181):208–88. 10.1016/j.resuscitation.2022.10.005 36336195

[5] Avila-Alvarez A Davis PG Kamlin COF Thio M. Documentation during neonatal resuscitation: a systematic review. Arch Dis Child Fetal Neonatal Ed. (2021) 106(4):376–80. 10.1136/archdischild-2020-319948 33243927

[6] Root L van Zanten HA den Boer MC Foglia EE Witlox R Te Pas AB. Improving guideline compliance and documentation through auditing neonatal resuscitation. Front Pediatr. (2019) 7:294. 10.3389/fped.2019.00294 31380327 PMC6646726

[7] Bjorland PA Ersdal HL Oymar K Rettedal SI. Compliance with guidelines and efficacy of heart rate monitoring during newborn resuscitation: a prospective video study. Neonatology. (2020) 117(2):175–81. 10.1159/000506772 32248187 PMC9533428

[8] Rettedal S Kibsgaard A Eilevstjonn J Kvaloy JT Bjorland PA Markhus Pike H Impact of immediate and continuous heart rate feedback by dry electrode ECG on time to initiation of ventilation after birth: protocol for a randomised controlled trial. BMJ Open. (2022) 12(9):e061839. 10.1136/bmjopen-2022-061839 36691167 PMC9454047

[9] Kibsgaard A Ersdal H Kvaloy JT Eilevstjonn J Rettedal S. Newborns requiring resuscitation: two thirds have heart rate >/=100 beats/minute in the first minute after birth. Acta Paediatr. (2023) 112(4):697–705. 10.1111/apa.16659 36607256

[10] Patterson JK Ishoso D Eilevstjonn J Bauserman M Haug I Iyer P Delayed and interrupted ventilation with excess suctioning after helping babies breathe with Congolese birth attendants. Children (Basel). (2023) 10(4):652. 10.3390/children10040652 37189901 PMC10137041

[11] Kolstad V Pike H Eilevstjonn J Buskov F Ersdal H Rettedal S. Use of pulse oximetry during resuscitation of 230 newborns—a video analysis. Children (Basel). (2023) 10(7):1124. 10.3390/children10071124 37508621 PMC10377843

[12] Pike H Kolstad V Eilevstjonn J Davis PG Langli Ersdal H Irene Rettedal S. Newborn resuscitation timelines: accurately capturing treatment in the delivery room. Resuscitation. (2024) 197:110156. 10.1016/j.resuscitation.2024.110156 38417611

[13] Perpetuini D Filippini C Cardone D Merla A. An overview of thermal infrared imaging-based screenings during pandemic emergencies. Int J Environ Res Public Health. (2021) 18(6). 10.3390/ijerph18063286 PMC8004954 33810086

[14] Brzezinski RY Rabin N Lewis N Peled R Kerpel A Tsur AM Automated processing of thermal imaging to detect COVID-19. Sci Rep. (2021) 11(1):17489. 10.1038/s41598-021-96900-9 34471180 PMC8410809

[15] Heimann K Jergus K Abbas AK Heussen N Leonhardt S Orlikowsky T. Infrared thermography for detailed registration of thermoregulation in premature infants. J Perinat Med. (2013) 41(5):613–20. 10.1515/jpm-2012-0239 23443261

[16] Adams AK Nelson RA Bell EF Egoavil CA. Use of infrared thermographic calorimetry to determine energy expenditure in preterm infants. Am J Clin Nutr. (2000) 71(4):969–77. 10.1093/ajcn/71.4.969 10731505

[17] Maurya L Zwiggelaar R Chawla D Mahapatra P. Non-contact respiratory rate monitoring using thermal and visible imaging: a pilot study on neonates. J Clin Monit Comput. (2023) 37(3):815–28. 10.1007/s10877-022-00945-8 36463541 PMC10175339

[18] Abbas AK Heimann K Jergus K Orlikowsky T Leonhardt S. Neonatal non-contact respiratory monitoring based on real-time infrared thermography. Biomed Eng Online. (2011) 10:93. 10.1186/1475-925X-10-93 22243660 PMC3258209

[19] Knobel RB Guenther BD Rice HE. Thermoregulation and thermography in neonatal physiology and disease. Biol Res Nurs. (2011) 13(3):274–82. 10.1177/1099800411403467 21586499 PMC3775585

[20] Engan K Meinich-Bache Ø Brunner S Myklebust H Rong C García-Torres J Newborn time—improved newborn care based on video and artificial intelligence—study protocol. BMC Digit Health. (2023) 1:10. 10.1186/s44247-023-00010-7

[21] García-Torres J Meinich-Bache Ø Brunner S Johannessen A Rettedal S Engan K. Towards using thermal cameras in birth detection. In: 2022 IEEE 14th Image, Video, and Multidimensional Signal Processing Workshop (IVMSP); 2022 Jun 26-29, Nafplio, Greece. Piscataway, NJ: IEEE (2022). 10.1109/IVMSP54334.2022.9816177

[22] Ersdal HL Mduma E Svensen E Perlman JM. Early initiation of basic resuscitation interventions including face mask ventilation may reduce birth asphyxia related mortality in low-income countries: a prospective descriptive observational study. Resuscitation. (2012) 83(7):869–73. 10.1016/j.resuscitation.2011.12.011 22198423

